# Being trusted: How team generational age diversity promotes and undermines trust in cross‐boundary relationships

**DOI:** 10.1002/job.2045

**Published:** 2015-09-01

**Authors:** Michele Williams

**Affiliations:** ^1^ Cornell UniversityIthacaNew YorkU.S.A.

**Keywords:** being trusted, boundary spanners, social categorization, age diversity, age heterogeneity, age composition

## Abstract

We examine how demographic context influences the trust that boundary spanners experience in their dyadic relationships with clients. Because of the salience of age as a demographic characteristic as well as the increasing prevalence of age diversity and intergenerational conflict in the workplace, we focus on team age diversity as a demographic social context that affects trust between boundary spanners and their clients. Using social categorization theory and theories of social capital, we develop and test our contextual argument that a boundary spanner's experience of being trusted is influenced by the social categorization processes that occur in dyadic interactions with a specific client and, simultaneously, by similar social categorization processes that influence the degree to which the client team as a whole serves as a cooperative resource for demographically similar versus dissimilar boundary spanner–client dyads. Using a sample of 168 senior boundary spanners from the consulting industry, we find that generational diversity among client team members from a client organization undermines the perception of being trusted within homogeneous boundary spanner–client dyads while it enhances the perception of being trusted within heterogeneous dyads. The perception of being trusted is an important aspect of cross‐boundary relationships because it influences coordination and the costs associated with coordination. © 2015 The Author Journal of Organizational Behavior Published by John Wiley & Sons Ltd

Individuals on knowledge‐intense projects must often gain the cooperation of counterparts over whom they have no hierarchical control (Adler, 2001). When these projects span organizational boundaries, the ability to develop interpersonal trust is particularly critical (Perrone, Zaheer, & McEvily, 2003). Trust enables cooperation when authority relationships are absent, reduces the need to monitor others' behavior, and facilitates access to “richer‐freer” information (Fulmer & Gelfand, 2012; McEvily, Perrone, & Zaheer, 2003; Ring & Van de Ven, 1994; Uzzi, 1997). Trust not only promotes knowledge exchange (Golden & Raghuram, 2010; Levin & Cross, 2004; Mäkelä & Brewster, 2009) but also may increase support for the boundary spanner and commitment to decisions made by the boundary spanner similar to the way that trust in a leader does (Brockner, Siegel, Daly, Tyler, & Martin, 1997; Dirks & Ferrin, 2002). Despite the potential benefits of trust, developing trust across boundaries can be difficult. People frequently perceive individuals from other groups and organizations as adversaries with aspirations, beliefs, or styles of interacting that threaten their goals (Kramer & Lewicki, 2010; Williams, 2001, 2007).

Developing trust with clients is further complicated because trust does not develop in a vacuum. Individuals must often build interpersonal trust within a broader team context of individuals who are from dissimilar demographic groups. Rousseau, Sitkin, Burt, and Camerer (1998), for instance, called for researchers to focus more on contextual influences on trust. Similarly, Burt and Knez (1996) argued that although interpersonal trust is often examined within a dyad, trust is most often built and maintained in the presence of an audience of “variably close friends, foes, and acquaintances” (p. 83). Despite these calls for investigating the influence of context in the study of trust, we still know little about how context influences trust development (Currall & Inkpen, 2006; Fulmer & Gelfand, 2012; Schoorman, Mayer, & Davis, 2007).

In this paper, we investigate how the age composition of a client team from another organization forms a context that may influence a boundary spanner's experience of trust in his or her dyadic client relationships. Specifically, we examine the extent to which age heterogeneity, operationalized as generational heterogeneity, affects team members' perceptions that they are trusted. Although our focus on generational age diversity and the perception of being trusted is fairly unique in the trust literature (Fulmer & Gelfand, 2012), there is growing scholarly interest in generational diversity and age‐related stereotypes (e.g., Finkelstein, King, & Voyles, 2014; Joshi, Dencker, Franz, & Martocchio, 2010; Smola & Sutton, 2002).

A generation is an identifiable group that shares birth years, significant life events at critical developmental stages, and often similar job values and attitudes (Joshi et al., 2010; Smola & Sutton, 2002). The presence of intergenerational conflict is increasingly being reported by HR professionals (SHRM, 2011), and age heterogeneity is becoming a significant organizational issue (Avery, McKay, & Wilson, 2007; Beatty & Visser, 2005; Fullerton & Toossi, 2001; Kunze, Boehm, & Bruch, 2011). Not only are workforce training, career development, and retention affected by age heterogeneity (Armstrong‐Stassen & Schlosser, 2011; Avery et al., 2007; de Lange et al., 2010; Goldberg, Finkelstein, Perry, & Konrad, 2004; Ng & Feldman, 2013) but so are team processes such as information sharing and helping (Chattopadhyay, 1999; Jehn, Northcraft, & Neale, 1999; Zenger & Lawrence, 1989). Further, as the U.S. workforce ages and both younger and older workers make up rising proportions of the workforce, most workplaces are becoming multigenerational (Joshi et al., 2010; Lyons & Kuron, 2014; Smola & Sutton, 2002) and this diversity may influence interorganizational relationships because age similarity has been found to influence relationships that cross organizationally relevant boundaries (e.g., Reagans, 2011; Zenger & Lawrence, 1989). Additionally, age diversity may influence relationships within interorganizational client teams both because age diversity is often more common than either gender or racial diversity in the upper echelons of organizations and also because age has been found to be highly salient and second only to race in the formation of friendship ties (Avery et al., 2007; McPherson, Smith‐Lovin, & Cook, 2001).

Thus, we develop and test the argument that a team's generational age composition will significantly moderate the relationship between dyadic age heterogeneity and perceptions of being trusted. We ask two questions: (1) “When dyad members are from different organizations, is age similarity in a dyad sufficient to lead a boundary spanner to believe that he or she is more trusted?” and (2) “If demographically similar dyad members do believe they are more trusted, does the generational age diversity of the members of the broader team influence a boundary spanner's perception of being trusted within a cross‐boundary dyad?”

With respect to dyadic relationships, the similarity‐attraction paradigm (Byrne, 1971) and social categorization theory (Hogg & Terry, 2000; Hornsey, 2008; Tajfel, 1981; Turner, 1987) suggest that demographic similarity may have a positive influence on trust because individuals from the same social category tend to view each other as more likeable and trustworthy than out‐group members (i.e., individuals from other categories; Brewer & Brown, 1998; Kramer, 1999). However, neither theory specifies the conditions under which team‐level heterogeneity may increase versus decrease perceived trust within demographically similar or dissimilar dyads (Joshi, Liao, & Roh, 2011). We contribute to the literature on trust by proposing and testing why a team's demographic composition forms a context that influences the perception of being trusted within cross‐boundary dyads embedded within that team.

We use the similarity‐attraction paradigm and social categorization theory to identify “bonding ties” or goodwill between boundary spanners and client team members and, thus, the network of goodwill available to dyads within the team context (Adler & Kwon, 2002; Kwon & Adler, 2014). We integrate social categorization theory at the dyadic level with the social capital or “goodwill” implications of social categorization at the team level to develop and test our contextual argument that a boundary spanner's experience of being trusted is influenced by the social categorization processes that occur in dyadic interactions with a specific client and, degree to which the client team as a whole serves as a cooperative resource full of goodwill for the dyadic boundary spanner–client relationship. We focus on cross‐boundary dyads as unique relationships that not only vary in their quality but also form an important component of “a system of interdependent dyadic relationships” (Avolio, Walumbwa, & Weber, 2009; Uhl‐Bien, 2006) for which interpersonal trust is highly relevant (Fulmer & Gelfand, 2012).

Specifically, we examine age heterogeneity at the team level as a moderator that sometimes facilitates and sometimes inhibits the perception of trust within cross‐boundary dyads embedded in client teams. We hypothesize that when social categorization processes work simultaneously at the dyadic and team levels, the demographic heterogeneity of a boundary spanners' team of clients forms a context that has the opposite influence on the perception of being trusted in demographically homogeneous versus heterogeneous interorganizational dyads.

As a phenomenon, boundary‐spanning dyads embedded within cross‐boundary teams occur in many types of organizational groups such as cross‐functional product development teams, professional service relationships, co‐commercialization agreements between start‐up and incumbent firms, and research and development alliances. We examine our hypotheses in the professional service setting of management consulting. Specifically, we investigate how a senior‐level consultant's perception of receiving interpersonal trust from one of his or her clients is influenced by the demographic context of his or her senior client team. All relational ties from the boundary spanners in our study to their clients on the client team extend across firm boundaries rather than across functional, departmental, or divisional boundaries. Figure 1 depicts the types of boundary spanner–team contexts we investigate in this paper. All individuals in our diagram have ties to one another. However, Figure 1 depicts only those that are critical for explaining our theoretical arguments.

**Figure 1:**
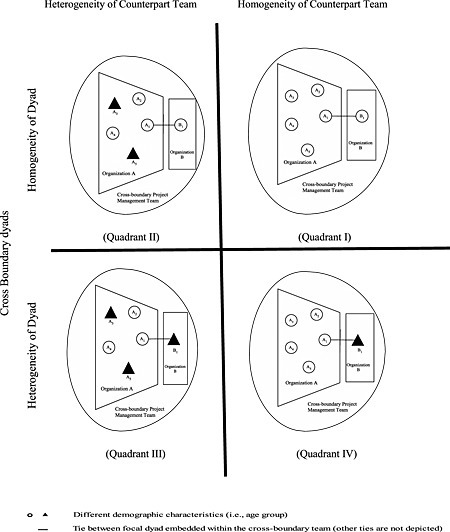
Boundary spanner, dyadic counterpart, and team demographic composition

This article is organized as follows. First, we define trust and discuss the relevance of the perception of being trusted in cross‐boundary teams. We then develop hypotheses about the relationship between team‐level demographic heterogeneity and the perception of being trusted in the dyads embedded in these client teams. In the following sections, we present our empirical results and then conclude with a discussion and implications.

## Being Trusted

Trust is defined as one's willingness to rely on another's actions in a situation involving the risk of opportunism or harm, thus making one vulnerable to the actions of another (Mayer, Davis, & Schoorman, 1995; Rousseau et al., 1998). Trust is based on an individual's expectations that others will behave in ways that are helpful or at least not harmful (Gambetta, 1988). These expectations, in turn, are based on people's perceptions of others' trustworthiness—i.e., their benevolence, integrity, and ability (e.g., Mayer et al., 1995).

### Perceptions of being trusted

Although trustworthy behavior is often observable, individuals also respond to one another based on their perception of being trusted (Salamon & Robinson, 2008). In this study, we investigate the impact of demographic composition on the *perception of being trusted*, that is, the perception that another individual is willing to rely on you given the risk of opportunism or harm. Lau and Lam (2007) found that “employees' perception of whether their supervisors trust them had a strong impact on their performance and attitudes, above and beyond the effects of whether employees trust their supervisor” (p. 1). In another study, they found that “when leaders felt more trusted, (their) teams showed more citizenship behavior” (Lau & Lam, 2008, p. 141). Lau, Lam, and Wen (2014) also found that perceptions of being trusted were related to organizational self‐esteem and individual performance. Similarly, Salamon and Robinson (2008) found that employees' perceptions of being trusted by management were related to sales performance.

Whereas the perception of being trusted is important across a variety of work relationships because it evokes norms of reciprocity (Lau et al., 2014), we argue that the perception of being trusted is particularly important in cross‐boundary relationships because it influences the ability to mobilize cooperation and reduces the cost of transacting.

For instance, the perception of low trust affects boundary spanners' ability to engage others in cooperative efforts. Although trust can mobilize cooperative behavior (Adler & Kwon, 2002; de Jong & Elfring, 2010; Kramer & Lewicki, 2010; McEvily et al., 2003), it can only be actively or consciously utilized if people perceive that they are trusted. A boundary spanner, for example, is only likely to mobilize this resource by suggesting a solution to a problem that requires others to share sensitive information if he or she believes that those individuals trust him or her enough to take this risk. However, mobilizing the resource of perceived trust can be critical because, if this perception is accurate, being trusted with sensitive information facilitates coordination, allows the transfer of unique external knowledge, and facilitates the receipt of useful tacit knowledge (Cummings, 2004; Currall & Judge, 1995; Levin & Cross, 2004). It also conserves resources by preventing costly searches to verify information from individuals who you believe do not trust you enough to accurately share sensitive information.

Furthermore, the perception of being trusted can increase an individual's ability to focus on core tasks (Mayer & Gavin, 2005). In contrast, when people believe that they are not trusted or think that they are under evaluative scrutiny, they dedicate time to ruminating over this lack of trust and are more likely to interpret ambiguous behaviors as sinister acts (Kramer, 1994). This misinterpretation of benign behaviors is likely to increase the use of costly safeguards by individuals who perceive that they are not trusted. Consequently, another benefit of the perception of being trusted is the “lack of engaging in self‐protective behaviors” (Mayer & Gavin, 2005, p. 876).

Finally, the perception of being trusted is likely to provide a good estimate of boundary spanners' feelings of inclusion. Although inclusion typically refers to experiences of employees within an organization, Pelled, Ledford, and Mohrman (1999, p. 1014) defined inclusion as “the degree to which an employee is accepted and treated as an insider by others in a work system.” When boundary spanners believe that others are willing to accept them by relying on them with tasks and information, they are likely to feel accepted in the context of that cross‐boundary relationship and able to contribute fully.

## Demographic Heterogeneity and Trust

In our context, boundary spanners attempt to build trust while surrounded by variably close and variably heterogeneous client team members (to paraphrase Burt & Knez, 1996, p. 83). However, trust research has not focused on the influence of team demography on the perception of trust in the dyadic relationships embedded within cross‐boundary or interorganizational teams (Currall & Inkpen, 2006; Fulmer & Gelfand, 2012). Moreover, with few exceptions (e.g., Lau & Murnighan, 2005), current research on demography says little about the influence of heterogeneous team environments on the homogeneous dyadic relationships that are embedded within those demographically diverse teams. In this section, we review literature on the influence of demographic heterogeneity on dyadic interpersonal trust. Then, we examine client team generational age heterogeneity as an inhibitor and facilitator of trust in dyads.

### Heterogeneity and trust

The psychological basis for the influence of demographic heterogeneity on trust stems both from social categorization theory (Tajfel, 1981; Turner, 1987) and from similarity‐attraction theory (Berscheid & Walster, 1978; Byrne, 1971). Social categorization theory suggests that people gain self‐esteem from positive perceptions of the groups to which they belong and associate liking and trust with members of those in‐groups (Brewer & Brown, 1998). Even research on groups formed in laboratories on the basis of trivial distinctions has consistently found that people associate liking and positive beliefs about trustworthiness with others who belong to the same arbitrarily assigned in‐group (Brewer & Brown, 1998; Hornsey, 2008).

Consistent with social categorization theory, similarity‐attraction theory predicts that demographic similarity increases interpersonal attraction and liking (Berscheid & Walster, 1978; Byrne, 1971). Both similarity‐attraction theory and social categorization theory suggest that dissimilar group membership is associated with lower positive affect or even the “absence of positive affect” (Brewer & Brown, 1998). Factors that influence liking and positive affect also influence trust (Jones & George, 1998), because positive feelings are affirming and heighten the motivation to believe that others trust you (Williams, 2001).

#### Age heterogeneity and trust

Although research on organizational demography rarely looks at trust specifically, it often investigates trust‐related outcomes such as communication and conflict (Williams & O'Reilly, 1998). For example, Zenger and Lawrence (1989) found that age diversity was negatively related to the frequency of technical communication within a group. Trust may have played a role in these communication levels because self‐disclosure and information sharing are risky cooperative actions (i.e., trusting behaviors; Currall & Judge, 1995). Chatman and Flynn (2001) found that greater demographic heterogeneity in teams initially resulted in norms stressing lower cooperation (i.e., less trusting behavior), and Flynn, Chatman, and Spataro (2001) found that demographic dissimilarity from co‐workers was negatively related to aggregated impressions of cooperativeness and the ability to accomplish assigned tasks. Thus, demographically heterogeneous dyads are likely to experience less trusting behavior (i.e., cooperativeness and information sharing) and, therefore, perceive that they are less trusted by their partner than members of homogeneous dyads.

#### Age heterogeneity in cross‐boundary relationships and trust

The effect of demographic similarity on individuals who span organizational boundaries is more complex because the out‐group effects of dissimilar organizational membership may outweigh the in‐group effects of age similarity. Conversely, the in‐group effects of being on the same project team may outweigh the out‐group effects of age dissimilarity. However, there are several reasons to believe that age similarity/dissimilarity will remain salient in cross‐boundary relationships. Not only is age a highly visible demographic category (Cleveland, Shore, & Murphy, 1997), it is an important driver of friendship relationships and subgroup development (Choi & Sy, 2010). Relationships based on age may be also be driven by that fact that stigmatizing age stereotypes still exist (Finkelstein et al., 2014; Hassell & Perrewe, 1995) and age, especially when associated with generational differences, is likely to covary with shared values and attitudes that exacerbate the negative effects of social categorization (Joshi et al., 2010; Kearney, Gebert, & Voelpel, 2009). For example, stereotypes exist that Baby Boomers are “resistant to change and technology,” Generation Yers “too informal and excessively tied to technology,” and Generation Xers “too independent” (AARP, 2007; SHRM, 2011). In addition, the fact that people are relatively comfortable with making ageist (vs. racist or sexist) comments may unite people of similar ages (Heslin, Bell, & Fletcher, 2012).

Even positive stereotypes of younger or older workers may not be sufficient to overcome conflicts because age paradoxes exist. For example, older workers are considered dependable and wise but “the rapidly changing nature of work means that the skills and knowledge of older workers may not be those that younger people (value or) need to develop” (Ranzijn, 2004, p. 287). Moreover, research suggests a “clear mismatch between the perceived value of the accumulated skills of older workers and the current and anticipated requirements of the rapidly changing world of work” (Ranzijn, 2004, pp. 284–285).

Several studies suggest relational benefits of age similarity within organizations (e.g., Kirchmeyer, 1995; O'Reilly, Caldwell, & Barnett, 1989) as well as effects of age similarity that span organizationally relevant boundaries (e.g., Reagans, 2011; Zenger & Lawrence, 1989). Zenger and Lawrence (1989), for example, found that age similarity was related to cross‐boundary communication. Specifically, members of different groups who were similar in age communicated more frequently with similarly aged others across project groups than with those who were more dissimilar in age. Reagans (2011) found that the positive effects of age similarity were amplified by physical proximity such as that created on colocated project teams. Thus, we propose that age homogeneity versus heterogeneity will influence the perception of being trusted in dyads that span organizational boundaries. We investigate this proposition in a context in which the dyad members, that is, the boundary spanner (B_1_, from firm B) and his or her dyadic client (A_1_, from firm A), are from different organizations (Figure 1).

Hypothesis 1 Boundary spanners in interorganizational dyads with clients who differ in age (heterogeneous dyads) will perceive less trust from their dyadic client counterparts than boundary spanners in age‐homogeneous dyads.

## Being Trusted in Dyads Embedded in Heterogeneous Client Teams

In this section, we investigate team‐level generational age heterogeneity as an inhibitor and facilitator of the trust experienced in the dyads embedded in client teams. We examine dyads consisting of a boundary spanner (B_1_, from firm B) and his or her dyadic client (A_1_, from firm A). We first focus on heterogeneous dyads embedded in homogeneous or diverse client teams from firm A and then examine homogeneous dyads that are also embedded in teams from firm A.

### Team heterogeneity as a source of social capital

Both social categorization theory and the similarity‐attraction paradigm form the psychological basis for “bonding” ties among similar individuals within groups or social networks. Bonding ties constitute social capital defined as “the goodwill available to individuals and groups” where goodwill includes positive feelings as well as “sympathy, trust and forgiveness” (Adler & Kwon, 2002: 18; Kwon & Adler, 2014: 412, respectively). Goodwill is a cooperative resource that has been associated with outcomes such as knowledge transfer (Maurer, Bartsch, & Ebers, 2011) and innovation (Obstfeld, 2005). Both social categorization theory and the similarity‐attraction paradigm suggest that similarly demographic group members can generate “good will” in terms of positive affect and perceptions of the trustworthiness of similar others (Brewer & Brown, 1998; Hogg & Terry, 2000; Hornsey, 2008). In teams, ties to demographically similar others are bonding ties that are likely to be associated with goodwill.

Further, within teams and organizations, trust and goodwill can be transferred (Burt & Knez, 1996; Ferrin, Dirks, & Shah, 2006). This process can occur in two ways. First, trust can be transferred through indirect communication (i.e., hearing or overhearing third‐party gossip; Burt & Knez, 1996). For example, a client team member may learn about boundary spanners' competent, helpful work or their discretion with sensitive matters, and this reputation may enhance their trust in those boundary spanners (Ferrin et al., 2006). Second, transferability can occur through observation, that is, by seeing how a particular individual is treated by others. For example, the excessive helpfulness with which women are sometimes treated decreases observers' perceptions of their competence and ability (and ability is a dimension of trustworthiness; Good & Rudman, 2010). Similarly, if team members observe a boundary spanner being treated with deference or, conversely, being ignored or interrupted, perceptions of the reputation, status, and trustworthiness of that boundary spanner may be influenced indirectly by these observations (Ridgeway & Erickson, 2000).

### Team heterogeneity as a facilitator of trust in heterogeneous dyads

In the following section, we integrate the social network paradigm (Burt & Knez, 1996; Ferrin et al., 2006; Reagans, 2011) with scholarship on trust, social categorization, and social capital in order to propose how bonding ties to demographically similar others on a client team affect the perceptions of boundary spanners in dyadic relationships that are embedded within broader project teams. We focus on heterogeneous dyads embedded in otherwise homogeneous versus heterogeneous client teams, quadrants IV and III of Figure 1. A heterogeneous dyad might consist of an independent, tech savvy Generation X consultant and an experienced, team‐oriented Baby Boomer client. The client team may consist of all Baby Boomers (homogeneous) or be a mix of Gen‐Xers and Baby Boomers (heterogeneous).

When a heterogeneous, Gen‐X/Boomer, dyad is embedded in a heterogeneous client team, the boundary spanner is demographically dissimilar from the dyadic counterpart and the remaining team of counterparts is diverse such that the boundary spanner (B_1_) will find similar others in the remaining team of client counterparts (e.g., other Gen‐Xers, A_3_ and A_5_, quadrant III). This stands in contrast to the case in which all client team members including the dyadic counterpart are similar to one another (e.g., A_1_ through A_5_ are Baby Boomers) and dissimilar from the boundary spanner (B_1_, a Gen‐Xer, quadrant IV).

A core assumption of our paper is that each boundary spanner–client dyad within a team is unique and that the varying quality of these relationships affects important interpersonal perceptions and coordination enhancing behaviors (Avolio et al., 2009; Uhl‐Bien, 2006). The second core assumption of our paper is that clients' perceptions of boundary spanners will be influenced by the perceptions of other client team members from the client organization. A significant body of work suggests that joint goals and a shared superordinate group membership such as similar organizational membership can improve the relationships between members of different demographic groups (Gaertner & Dovidio, 2000; Hewstone, Rubin, & Willis, 2002). Thus, the influence of team members from one's own organization may be stronger when those members are also demographically similar. However, shared organizational membership and common project goals are likely to be associated with some level of trust and influence between demographically dissimilar team members from the same organization. Based on past research that has found age effects in contexts with shared goals (Reagans, 2011; Zenger & Lawrence, 1989), we believe that effects of age similarity/dissimilarity on cross‐boundary projects are likely and require empirical examination.

#### Increased trust

We propose that trust transferability may lead to increased trust by a demographically dissimilar dyadic client counterpart (A_1_) embedded in a heterogeneous client team. First, social categorization theory (Brewer & Brown, 1998; Hogg & Terry, 2000; Hornsey, 2008; Tajfel, 1981; Turner, 1987) suggests that client team members from the client organization (A_3_ and A_5_, quadrant III) who are demographically similar to the boundary spanner (also Gen‐Xers), may hold higher perceptions of the boundary spanner's trustworthiness and feel more positive affect for that demographically similar boundary spanner (B_1_, quadrant III) than they would for a demographically dissimilar boundary spanner. Research on generations further suggests that age similarity may also be associated with shared values and job attitudes (Joshi et al., 2010), and value congruence has been linked to trust (Edwards & Cable, 2009).

The dyadic client counterpart (A_1_) of that boundary spanner, a Boomer, may be positively influenced by team members A_3_ and A_5_ (quadrant III) who share a common organizational membership with A_1_ but are demographically similar to the boundary spanner (a Gen‐Xer) and may hold and express positive perceptions of the boundary spanner's (B_1_'s) trustworthiness in terms of B_1_'s ability and shared values that form the basis for integrity (i.e., positive third‐party ties, gossip, and treatment; Burt & Knez, 1996; Labianca & Brass, 2006).

Ferrin et al. (2006) have argued that individuals “use third‐party information to supplement their own direct information because of the difficulties of making trust judgments on the basis of ambiguous and incomplete information” (p. 875). Because interorganizational projects, especially knowledge‐based projects, are characterized by ambiguity, complexity, and incomplete information (Adler, 2001; Perrone et al., 2003; Ring & Van de Ven, 1994), we expect clients to use the presence and absence of other team members' trust ties toward the boundary spanner to inform their own level of trust in the boundary spanner and, thereby, the boundary spanner's perception of being trusted. In addition, these ties are evidenced through third‐party gossip and interpersonal treatment, which can transfer trust (Burt & Knez, 1996). In our case, each other member of A_1_'s team may or may not have a “bonding” trust tie to the boundary spanner B_1_ (a Gen‐Xer) and, thus, may transfer their trust or lack of trust in boundary spanner (B_1_) to the relationship between A_1_ and B_1_.

We argue that through social categorization processes, generational similarity (dissimilarity) will influence the formation of trust ties between the boundary spanner and each client team member. This pattern of ties will influence the boundary spanner's perception of being trusted in a specific dyadic relationship. For simplicity and parsimony in the following illustrative example, we have adopted Ferrin et al.'s (2006) empirically grounded, social network notation of 1 for a trust tie and 0 for an absent or negative trust tie. Our example follows.

In quadrant IV, each team member (A_2_ though A_5_) is demographically dissimilar from the boundary spanner (all are Boomers) and may lack a trust tie to him or her, thus transferring zero trust ties to the relationship between A_1_ and B_1_. In quadrant III, team members A_3_ and A_5_ are demographically similar to the boundary spanner and may each have a trust tie to him or her, thus transferring two trust ties. Even if the transferred trust ties of A_3_ and A_5_ (out‐group Gen‐Xers) do not carry equal weight to one another or to the lack of ties of A_2_ and A_4_ (in‐group Boomers), it is still likely that when a Boomer client is in a heterogeneous team (quadrant III with two trust ties from the other team members to the boundary spanner), he or she will have a higher level of trust in a Gen‐X boundary spanner than when that Boomer is in a homogeneous client team (quadrant IV with zero trust ties) and that the Gen‐X boundary spanner will have the perception of being more trusted in the heterogeneous client team.

Hypothesis 2 Boundary spanners (B_1_) in dyadic relationships with clients who are dissimilar in age will perceive more trust from a dyadic client counterpart (A_1_) when the dyad is embedded in a team of clients who are heterogeneous (quadrant III) versus homogeneous in age (quadrant IV) (see Figure 1).

### Team heterogeneity as an obstacle to trust in homogeneous dyads

In this section, we focus on homogeneous dyads embedded in homogeneous versus heterogeneous client teams, quadrants I and II of Figure 1. When a homogeneous dyad of two Boomers, for example, is embedded in a homogeneous client team, all client team members including the dyadic counterpart are similar to one another (A_1_ through A_5_, all Boomers) and similar to the boundary spanner (B_1_, also a Boomer, quadrant I). This stands in contrast to the case of a homogeneous dyad embedded in a heterogeneous client team in which the boundary spanner is demographically similar to his or her dyadic counterpart, but the remaining team of clients is diverse such that the boundary spanner (B_1_) will find *fewer* “bonding ties” to demographically similar others in the remaining team of clients because the team has a mix of Boomers and Gen‐Xers (quadrant II).

#### Reduced trust

Social categorization processes may cause reduced reliance or trusting behavior by a demographically similar dyadic counterpart (A_1_) in a manner that is the flip side of the social categorization processes in the previous section. First, social categorization theory (Brewer & Brown, 1998; Hogg & Terry, 2000; Hornsey, 2008; Tajfel, 1981; Turner, 1987) suggests that as out‐group members, demographically dissimilar team members from the client organization (A_3_ and A_5_, quadrant II, Gen‐Xers) may hold lower perceptions of a boundary spanner's trustworthiness and feel less positive affect for that demographically dissimilar boundary spanner (B_1_, quadrant II, Boomer) than they would for a demographically similar boundary spanner. A client in a homogeneous dyad (A_1_, quadrant II) may come to perceive the boundary spanner as inherently less trustworthy and competent through interactions with others from his or her own organization who are demographically dissimilar from the boundary spanner. For example, Gen‐Xers, A_3_ and A_5_ (quadrant II), may view the boundary spanner, a Boomer, as averse to using cutting edge technology, as placing too high a value on team cohesion and as placing too little value on risk taking and, therefore, perceive B_1_ as less trustworthy in terms of ability and in terms of acting in accordance with *acceptable* values. These views, in turn, may influence A_1_ (i.e., negative third‐party ties; Burt & Knez, 1996; Labianca & Brass, 2006).

We argue that generational differences and age stereotypes (Finkelstein et al., 2014) can influence perceptions of the core dimensions of trustworthiness, in particular ability and integrity (Colquitt, Scott, & Lepine, 2007; Mayer et al., 1995). Generational differences are often associated with differences in expertise or ability (Hewlett, Sherbin, & Sumberg, 2009). However, generational differences are also associated with conflicting values (Dencker, Joshi, & Martocchio, 2007; Hewlett et al., 2009), and these conflicting values make perceptions of integrity relevant. Behaving with integrity requires individuals to hold and act upon “acceptable” or shared values (Mayer et al., 1995; Sitkin & Roth, 1993; Sitkin & Stickel, 1996). Thus, perceived integrity has an interpersonal requirement (i.e., the target is acting in accordance with his or her own values) and an interpersonal requirement (i.e., the perceiver also finds those values acceptable). Consistent with the interpersonal aspect of perceived integrity, value congruence has been linked to trust (Edwards & Cable, 2009) and value incongruence to a perceived lack of integrity and distrust (Sitkin & Roth, 1993; Sitkin & Stickel, 1996). Therefore, perceptions of generational differences in “acceptable” values related to team work, risk taking, self‐reliance, and work commitment, for example, are likely to influence perceptions of integrity and trustworthiness.

Applying Ferrin et al.'s (2006) findings about trust transferability to our example, each team member (A_2_ though A_5_) may connect to the boundary spanner through a “bonding” trust tie and thereby transfer their trust in the boundary spanner to A_1_. In quadrant I, each team member (A_2_ though A_5_) is demographically similar to the boundary spanner (all are Boomers) and each may transfer a trust tie, thus transferring four trust ties. In quadrant II, team members A_2_ and A_4_ are demographically similar to the boundary spanner and may have a trust tie to him or her, thus transferring two trust ties. However, Gen‐X team members A_3_ and A_5_ are demographically dissimilar from the boundary spanner and may lack a trust tie to him or her, thus transferring zero trust ties to the relationship between A_1_ and B_1_. Even if those absent trust ties are not given equal weight to the trust ties of the other Boomers, A_2_ and A_4_, it is still likely that A_1_ in a heterogeneous team (quadrant II, two trust ties from the other team members to the boundary spanner) will have a lower level of transferred trust in the boundary spanner than if A_1_ were in the homogeneous client team (quadrant I, four trust ties) and that the Boomer boundary spanner will have the perception of being less trusted in the heterogeneous team.

In addition, even if the dyadic counterpart (A_1_) of a boundary spanner were to discount the lack of trust ties of dissimilar team members from his or her organization to the boundary spanner and retain positive perceptions of B1's trustworthiness—for example, after determining that Gen‐Xers, A_3_ and A_5_, are ageist (quadrant II), the social categorization‐based lack of trust ties and goodwill from Gen‐Xers A_3_ and A_5_ to the boundary spanner could still influence A_1_'s *behavior*. The lack of goodwill and social capital from Gen‐Xers A_3_ and A_5_ could impair B_1_'s ability to mobilize the cooperation necessary to accomplish tasks, thereby making reliance on B_1_ unwise. Specifically, A_1_, a Boomer, may believe that social categorization processes have led other team members to stereotype the boundary spanner and perhaps act in a discriminatory or ageist manner toward the boundary spanner (B_1_). The dyadic counterpart (A_1_) might still view the boundary spanner (B_1_) as trustworthy—that is, competent, benevolent, and full of integrity. However, the boundary spanner (B_1_) would not have the goodwill or social capital to mobilize the cooperation of dissimilar, Gen‐X client team members from the client organization. Therefore, the dyadic counterpart (A_1_) might choose not to rely on the boundary spanner (B_1_) for reasons external to his or her beliefs in that boundary spanner's characteristic trustworthiness. This second process also rests on the dyadic and team‐level influences of social categorization on the social capital resources associated with the pattern of trust ties from team members to the boundary spanner.

As an illustration, a boundary spanner's counterpart (A_1_ quadrant II), who is demographically similar, may be unwilling to rely on the boundary spanner (B_1_) to resolve high‐tech problem “alpha” *not* because the boundary spanner (B_1_, a Boomer) is dispositionally untrustworthy—that is, lacking in benevolence, adherence to acceptable values (integrity), or technical ability—but rather because the Gen‐X team members lack trust and goodwill toward Boomers, who they view as less technologically savvy (ability) and as placing insufficient value on risk taking and innovation (lacking acceptable values). The boundary spanner (B_1_), however, is unlikely to feel trusted because he or she was not assigned problem “alpha” (i.e., not relied upon). B_1_ will still have the experience of being less trusted than a boundary spanner who is relied upon.

This type of disruption in trusting behavior represents a unique external risk to relying on the boundary spanner (Mayer et al., 1995). Mayer et al. (1995) stated, “In our model, the perception of [external] risk involves the trustor's belief about likelihoods of gains or losses outside of considerations that involve the relationship with the particular trustee” (Mayer et al., 1995: 726). In our case, the external risk stems from social categorization processes and resultant lack of goodwill‐based social capital that is beyond the control of the boundary spanner and outside of A_1_'s relationship with the boundary spanner. The boundary spanner (B_1_) is not responsible for the biases of the other team members (e.g., A_3_ and A_5_, quadrant II), but his or her ability to perform in a trustworthy manner is nonetheless affected by the goodwill available in this external, social context.

Hypothesis 3 Boundary spanners (B_1_) in dyads whose members are homogeneous in age will perceive less trust from their dyadic client counterpart (A_1_) when the dyad is embedded in a team of clients that are heterogeneous (quadrant II) versus homogeneous in age (quadrant I) (see Figure 1).

## Methods

### Industry context

Many types of interorganizational alliances such as technology collaborations, client–professional service firm relationships, research and development agreements, and co‐commercialization alliances involve interdependent knowledge‐intense projects. The framework developed here is applicable to all of these cases. However, empirically, we chose to examine boundary spanners from the professional service industry working with client teams because building trust with clients is especially important for boundary‐spanning consultants.

Consultants work on interdependent, knowledge‐intense projects consisting of non‐routine tasks that make trust beneficial (Seabright, Levinthal, & Fichman, 1992). In addition, they gain career rewards for developing strong trust‐based relationships that lead to future work (Maister, 1997). Thus, the importance of trust for management consultants suggests that they are likely to be more motivated than individuals in other industries to overcome obstacles to building trust with counterparts. Consultants should be less susceptible to the proposed negative influences of demographic heterogeneity and, as a category of boundary spanners, should provide a strong test of this theory.

### Sample

Surveys were distributed to 250 senior‐level consultants from one of the top 10 international management consulting firms headquartered in the U.S.A. We received 227 participant surveys for a 91 percent response rate. After eliminating surveys with missing data, we obtained a final sample of 191 for the dyadic analyses. Because some consultants were working on small projects with only one counterpart (i.e., no team), the sample size for the team analyses was 168. The final sample did not differ significantly from those receiving surveys on demographic characteristics. The average age of participants was 40 years with an average firm tenure of 7 years. Eighty‐five percent had an MBA or other graduate degree. Nine percent were women, which reflected the gender balance of the firm at the senior level. Thirty‐six percent were European.

Because our participant boundary spanners (B_1_) were working on different client teams, we have 189 unique B_1_s rating A_1_s on the variables of interest. We thus have 189 independent observations.

Specifically, our client teams reflected the non‐hierarchal decision‐making unit for the project. Just as members of the top management team of an organization generate and oversee the implementation of strategy for an entire organization, the project management team for a consulting project generates and oversees the content, strategy, and implementation process for the consulting project. Our client team included the key client decision makers from the client organization—decision makers who oversee the goals, budget, and other strategic level decisions related to the project. Although our client teams were themselves relatively non‐hierarchical, the consultant and each client team member typically had a staff of subordinates who assisted them in implementing the decisions of the team.

### Procedure

We surveyed participants of an in‐house (company designed and implemented) 1‐week professional development seminar. Eleven separate but equivalent seminars were held at remote locations, and 20 to 40 consultants participated in each of the eleven seminars, the last of which was held in December 2001. Participants were given a dedicated half‐hour block to fill out the survey. Ten categorical “dummy” variables for survey administration sessions 2–11, with the first session as the referent session, yielded non‐significant results and were excluded from the analyses.

### Survey format

The survey consisted of two sections: a project section and a perceived dyadic‐relationship section. The project section included questions about a boundary spanner's (B_1_, Figure 1) current project size and network measures designed to capture the general interpersonal environment of the client team surrounding each boundary spanner. This section collected information about client counterparts (A_1_–A_5_, Figure 1) from two different projects and was formatted as follows. First, drawing extensively on the name‐generating questions used by Podolny and Baron (1997), we developed a client team name‐generating question that asked respondents for the first names or initials of senior‐level clients on each of their current client teams. In response to the generator, respondents could list up to five names per client team. Respondents, who provided the names of five client counterparts, were asked to estimate the number of additional contacts on that project who would meet the criteria of the name‐generating question. Eight‐eight percent (88%) of respondents reported that they had five or fewer client team members who were responsible for the decision‐making functions of the project. The average number was between three and four client team members serving this function (3.66, Table 1).

**Table 1:** Correlation matrix.

		Mean	*SD*	1	2	3	4	5	6	7	8	9	10	11	12	13	14	15	16	17	18
1	Boundary spanner (B_1_) perception of being trusted by dyadic counterpart (A_1_) (perceived information sharing)	5.86	0.98	0.85																	
2	Boundary spanner (B1) perception of being trusted by dyadic counterpart (A_1_) (perceived reliance)	5.77	0.93	0.65**	0.79																
3	Emotional closeness to dyad partner	5.36	0.86	0.33**	0.40**	0.81															
4	Dyadic age heterogeneity	0.42	0.50	−0.22**	−0.20**	−0.20†	–														
5	Team age heterogeneity (*SD*)	0.27	0.29	−0.13†	−0.14†	−0.10	0.28**	–													
6	Average age group of team	2.05	0.29	0.08	0.12	0.02	0.20**	0.12	–												
7	Boundary spanner (B_1_'s) age	39.87	7.13	0.16*	0.16*	0.07	−0.20*	−0.01	0.01	–											
8	Boundary spanner (B_1_'s) gender	0.09	0.29	−0.15†	−0.16*	0.04	0.03	0.12	−0.05	−0.04	–										
9	Boundary spanner (B_1_'s) nationality	0.36	0.48	0.00	−0.08	0.00	0.25**	0.22**	0.12	−0.21**	−0.02	–									
10	Dyad partner (A_1_'s) age group	2.15	0.43	0.00	0.04	−0.10	−0.40**	0.35**	0.68**	−0.08	0.04	0.11	–								
11	Dyad partner's (A_1_'s) gender	0.11	0.31	0.10	0.20*	0.01	−0.20*	−0.21**	−0.03	0.05	−0.04	−0.14†	−0.12	–							
12	Relationship duration	2.09	2.86	0.12	0.17*	0.36**	−0.10	0.04	−0.02	0.29**	0.04	−0.09	0.03	−0.10	–						
13	Interaction frequency of dyad	3.51	1.38	0.07	−0.13†	−0.30**	−0.00	0.19*	−0.08	0.12	0.10	−0.07	0.04	0.01	0.15*	–					
14	Job level (B_1_)	0.30	0.46	0.08	0.00	0.04	−0.10	0.04	0.12	0.12	0.02	0.03	0.14†	−0.10	0.08	0.05	–				
15	Firm tenure (B_1_)	6.71	4.00	−0.03	−0.09	−0.00	−0.10	−0.02	−0.09	0.02	0.10	−0.27**	−0.03	0.02	0.23**	0.13	0.00	–			
16	Industry experience (B_1_)	6.80	7.09	0.13	0.22**	0.11	−0.10	−0.03	0.06	0.63**	−0.06	−0.12	−0.02	0.12	0.11	0.06	0.03	−0.30**	–		
17	Project management team size (B_1_)	3.66	1.12	0.16*	0.14†	0.19*	−0.10	0.14†	−0.08	−0.16*	0.06	0.09	0.02	−0.10	0.14†	0.02	0.13†	−0.05	−0.03*	–	
18	Division (B_1_) (1 = larger; 2 = smaller)	1.28	0.45	0.02	0.09	0.11	−0.20*	−0.07	−0.10	0.56**	0.13†	−0.44**	−0.13	0.08	0.38**	0.24**	−0.12	0.42**	0.31**	−0.18*	–
19	Project (B_1_) (1 = primary, 2 = secondary)	1.44	0.50	−0.02	−0.14†	−0.20*	−0.10	−0.01	−0.07	0.27**	0.02	0.02	−0.17*	0.00	0.11	0.19*	0.03	0.08	0.09	−0.20*	0.17*

† *p* < .1;

* *p* < .05;

** *p* < .01.

Next, respondents were randomly assigned to answer several questions about the size and duration of one of the two projects and provide specific information about their counterparts on that project. Slightly more than half of the consultants provided this information about the first project listed (55%), because some consultants did not have a second project. Respondents reported the age group of each client whom they had identified on that project (A_1_–A_5_).

Section two of the survey focused on the dyadic relationship between the boundary spanner (B_1_) and one client counterpart (A_1_, i.e., the first client named on the name generator). Asking all of the boundary‐spanning consultants (B_1_) to answer questions with respect to the first counterpart named simplified the verbal directions, eliminated the uncertainty, distrust, and discussion that might arise otherwise, and also eliminated embarrassment for people working on smaller engagements who did not have more than one senior‐level counterpart. This section contained the multi‐item measures described later.

### Measures: Dependent variables

#### Perception of being trusted

Consistent with Lau et al. (2014), we used two different dimensions of being trusted, behavioral reliance and disclosure (i.e., information sharing). For both dimensions, we captured responses using a seven‐point Likert scale (ranging from 1 = strongly disagree to 7 = strongly agree).

##### Behavioral reliance (dimension of the perception of being trusted)

The perception of reliance was measured with a four‐item behaviorally oriented recall measure. These trust items drew on Mayer and Davis' (1999) measure of trust and on Currall and Judge's (1995) measure of trust, the surveillance/distrust subdimension (surveillance items were adapted and reverse coded). Items included the following: (1) “This person feels comfortable giving me a problem that is critical to him/her,” (2) “This person lets me have a great deal of influence on issues that are important to him/her,” (3) “This person feels confident that results will follow from our discussions,” and (4) “This person doesn't like to depend on me to handle issues that are important to him/her (reverse scored)” (Cronbach's alpha = .79).

##### Information sharing (dimension of the perception of being trusted)

The perception of receiving information was measured with a four‐item behaviorally oriented recall measure. These trust items drew on Currall and Judge's (1995) measure of trust (the information sharing dimension). Items included the following: (1) “When we discuss important matters, this client shares his/her thoughts with me,” (2) “This individual gives me relevant information about important issues,” (3) “This person minimizes the amount of information he/she gives to me (reverse scored),” and (4) “This person lets me know what he/she thinks about key issues” (Cronbach's alpha = .85).

### Measures: Independent variables

#### Team age heterogeneity (client team, A_1_ to A_5_)

Team age heterogeneity was measured using a standard deviation measure as appropriate for our conceptualization of age diversity as variation and our use of social categorization theory: √[Σ(S*i* − Smean)^2^ / *n*] (Harrison & Klein, 2007, p. 1210). Consultants reported the age of each member of the client team using broad age ranges that translated into the following birth years and generations: 1 = born 1965–1981, Gen‐X; 2 = born 1945–1964, Boomer; and 3 = born 1929–1944, Traditionalist. Using our standard deviation measure, team age homogeneity equaled zero and the maximum value possible for this variable was 1 (Harrison & Klein, 2007). Although less appropriate for our research question, we also conducted robustness tests using other methods of operationalizing team heterogeneity: dummy coding (0 = homogeneous team, 1 = heterogeneous team), Blau's index, and a Euclidean distance measure (Harrison & Klein, 2007). The results of analyses using these different measures of team age heterogeneity yielded comparable results in terms of sign, significance levels, and effect size. Because our analyses use three levels of coding for age, which embeds the implicit assumption that differences between Traditionalists and Gen‐Xers is greater than the distance between Gen‐Xers and Boomers, we have provided full results using dummy coding, which does not require this assumption (Table A1, Appendix).

We used the three broad age categories mentioned earlier for two main reasons. First, our broad age categories separate our data by generations. Although there is disagreement about the exact dates, our dates are consistent with those most commonly used in the literature, “Swingers [or Traditionalists] (1934–1945), Baby Boomers (1946–1964), Generations X‐ers (1965–1977) …” (Smola & Sutton, 2002, p. 371). Generational differences are important for this study because they are associated with differing work values, which can lead to lower trust across generations. For example, relative to Gen‐Xers, Boomers felt more strongly that “work should be one of the most important parts of a person's life” and agreed less with the statement—“I would quit my job if I inherited a lot of money.” (Smola & Sutton, 2002, pp. 376–377). Despite the mixed research findings about specific differences across generations (Joshi et al., 2010; Lyons & Kuron, 2014), perceptions that these differences exist are widespread (Finkelstein, Ryan, & King, 2013; Finkelstein et al., 2014), and articles reinforcing these stereotypes appear regularly in the popular press (e.g., AARP, 2007; Hewlett et al., 2009). The existence of these stereotypes is important because the perceptions of such differences reinforce social categorization‐based processes.

Second, research indicates that team members' perceptions of their team's age composition are reliable and valid for *wide* age ranges. McPherson and Rotolo (1995) used a multi‐trait‐multi‐method design to compare group members' perceptions of age composition with perceptions of group leaders and direct observation. Their results showed that the relationship between a team's age composition and respondents' perceptions of the group's age composition ranged from .81 to .88 (.84 for team members, .88 for team leaders, and .81 for direct observation). They concluded that although each type of measurement introduced some degree of bias, the three methods were reliable substitutes for one another because controlling for method effects did not substantially change the reliability of the measures (.8 to .9).

#### Dyadic age heterogeneity

Dyadic age heterogeneity was captured using a categorical dummy variable (0 = homogeneous dyad, 1 = heterogeneous dyad). Boundary‐spanning consultants (B_1_) reported the age of each member of the client team (A_1_ to A_5_) using broad age ranges that translated into the following birth years and generations: 1 = born 1965–1981, Gen‐X; 2 = born 1945–1964, Boomer; and 3 = born 1929–1944, Traditionalist. They reported their own age in years, which was then coded into the appropriate generational age range for comparison with the range of their client dyadic counterpart (A_1_). Our boundary spanners were 23 percent Gen‐Xers, 73 percent Boomers, and 6 percent Traditionalists. Our dyads and client teams had considerable variance. When both dyads and teams were categorically coded as homogeneous or heterogeneous, our boundary spanner–client dyads were 57 percent homogeneous and 43 percent heterogeneous. Of the homogeneous dyads, 61 percent were associated with homogenous client teams and 39 percent were associated with heterogeneous client teams. Of the heterogeneous dyads, 38 percent were associated with homogenous client teams and 62 percent were associated with heterogeneous client teams. Our results held constant or were strengthened when we controlled for boundary spanners on the border of our generational cutoffs, who may have felt more similar to individuals in the following or previous generation. We included these controls in all of our analyses.

### Measures: Control variables

#### Relationship duration (dyadic)

The duration of the interpersonal relationship between the boundary‐spanning consultant (B_1_) and his or her client dyadic counterpart (A_1_) was reported by the boundary spanner in years. Because trust develops over time (Ring & Van de Ven, 1994), relationship duration is likely to be positively related to trust. Relationship duration may also function as a control variable for the demographic variables because some research has found that over time, the negative effects of group‐level diversity may fade (Chatman & Flynn, 2001).

#### Interaction frequency (dyadic)

The frequency of face‐to‐face interactions between the consultant (B_1_) and his or her client dyadic counterpart (A_1_) was reported by the boundary spanner using a six‐point Likert scale. Scale anchors represented a decreasing frequency of face‐to‐face interaction (1 = daily to 6 = less often than once a month), and thus, the measure was reverse coded. Chatman, Polzer, Barsade, and Neale (1998) found that demographically dissimilar individuals tended to spend less time in face‐to‐face interactions than demographically similar individuals.

#### Emotional closeness (dyadic)

Emotional closeness was measured with Williams and Polman's (2015) two‐item measure. Items included the following: “I like this person” and “I feel emotionally close to this person” (Cronbach's alpha = .81). Emotional closeness is a correlate of trust (Jones & George, 1998). Controlling for emotional closeness better isolates the perception of being trusted, from a more affect‐based measure. It may also function as a control variable for the demographic variables because the negative effects of group‐level diversity on group processes may fade over time (e.g., Chatman & Flynn, 2001), and this effect may be the result of developing emotionally closer personal relationships with others.

#### Demographic characteristics of the focal consultant

We controlled for the consultant's (B_1_'s) age, gender, nationality, firm tenure, job level, division membership, years of industry experience prior to consulting, and project importance because they are factors that may influence others' trust in a consultant. We controlled for division membership because the divisions differed significantly in their clients, culture, and approach at the time of the study and in subsequent years legally separated into two separate firms.

Each consultant's job level was provided by the firm. Consultants were asked to report all other variables. A categorical variable was constructed for the focal consultant's gender (0 = male; 1 = female) and job level (0 = one promotion away from partner; 1 = new partner, i.e., promoted to partner during the previous year). A categorical variable was constructed for nationality (0 = US, 1 = European). A categorical variable was constructed for the division for which the focal consultant worked (1 = larger division with short‐term projects, 2 = smaller division with longer‐term projects). Consultants' age and amount of industry experience prior to joining the consulting profession were operationalized in years. A categorical variable was constructed for project importance (1 = primary project, 2 = secondary project). Many of the control variables in our analyses were not statistically significant. Removing any or all of the non‐significant control variables did not alter our findings.

#### Demographic characteristics of the client (dyadic counterpart, A_1_) and client team

Project team size equaled the number of clients on the focal consultant's team of clients who were responsible for the decision‐making functions of the project. We controlled for size because it is likely to be easier to build trust with fewer people.

We controlled for the client's (A_1_'s) age group and gender. Nationality data were not available for the client team members (A_1_–A_5_). A categorical variable was constructed for the focal client's gender (0 = male; 1 = female) and for the client team's gender composition (0 = all male, 69% of the teams and 1 = mixed gender). Counterpart age was measured using the three broad generational age categories described previously. When dyads are heterogeneous, controlling not only for the age of the consultant and for the age group of the dyadic client counterpart but also for the average age group of the client team adjusts for the possibility that the results are driven by younger consultants and/or older clients. We also ran supplemental analyses with interactions between the consultant's age and the following variables: the average age of the team, dyadic age heterogeneity, and team age heterogeneity. These interactions were not significant. Moreover, the positive interaction between team heterogeneity and dyadic heterogeneity predicted by Hypothesis 3 is inconsistent with pervasive lower trust in younger, less experienced consultants.

Table 1 shows the means, standard deviations, correlations, and reliability estimates (calculated as Cronbach's alphas) for all variables in the analyses. The reliability scores for our multi‐item measures all exceeded the .70 criterion suggested by Nunnally (1978).

### Analyses

#### Moderation

We used regression analysis (ordinary least squares) to test our hypotheses. Hypotheses 2 and 3, which contained interaction effects, were tested using Equation (1). This equation shows the linear regression model for predicting *Y* from *D*, *T*, and the interaction between *D* and *T*, where *D* represents dyadic age heterogeneity, *T* represents team age heterogeneity, and the *D* • *T* product term represents the moderating effect of *T* (Aiken, West, & Reno, 1991). *C* represents the control variables.

(1) $$Y=\beta_{0}+\beta_{1}D+\beta_{2}T+\beta_{3}D•T+\beta_{c}C+\varepsilon$$

Because *D* is a categorical “dummy” variable (coded 0, 1), the intercept estimate for homogeneous dyads is *β*_0_ and for heterogeneous dyads is *β*_0_ + *β*_1_. The term *β*_1_ reflects the difference between the intercepts for the two groups (i.e., the distance between the regression lines when team composition is homogeneous, i.e., team heterogeneity, *T* = 0; Aiken et al., 1991)_._ The slope estimate for homogeneous dyads is *β*_2_ and for heterogeneous dyads is *β*_2_ + *β*_3_ (Aiken et al., 1991).

## Results

### Regression results

The results for Hypothesis 1 appear in Table 2 (columns 1 and 2). Hypothesis 1 predicted that boundary spanners in interorganizational dyads with clients who differ in age (heterogeneous dyads) would perceive less trust from their dyadic client counterparts than boundary spanners in age‐homogeneous dyads. After controlling for relationship duration, emotional closeness, and interaction frequency, Hypothesis 1 was supported. Dyadic age heterogeneity was negatively related to our two measures of the perception of being trusted (perceived information sharing, *b* = −0.40, *p* < .05, 95% CI −0.72 ≤ *b* ≤ −0.08; perceived reliance, *b* = −0.30, *p* < .05, 95% CI −0.59 ≤ *b* ≤ −0.01). These analyses controlled for boundary spanners whose ages were on the border of our generational cutoffs, those who may perceive themselves to be more similar to individuals from another generation. Adding team‐level control variables to these analyses did not alter our findings.

**Table 2:** Demographic composition and boundary spanner's perception of being trusted.

	Boundary spanner (B_1_'s) perceptions of being trusted by dyadic counterpart (A_1_)			
	1	2	3	4
	Perceived information sharing	Perceived reliance	Perceived information sharing	Perceived reliance
	H1	H1	H2, H3	H2, H3
Dyadic age heterogeneity	**−0.40** * (0.16)	**−0.30** * (0.15)	−0.60** (0.22)	−0.48* (0.20)
Emotional closeness	0.52** (0.09)	0.39** (0.08)	0.36** (0.09)	0.31** (0.09)
Boundary spanner's (B_1_'s) age	0.02 (0.02)	0.00 (0.02)	0.02 (0.02)	0.00 (0.02)
Boundary spanner's (B_1_'s) gender	−0.53* (0.23)	−0.41† (0.20)	−0.32 (0.23)	−0.33 (0.21)
Boundary spanner's (B_1_'s) nationality	0.01 (0.16)	−0.10 (0.15)	0.08 (0.16)	−0.02 (0.16)
Dyadic counterpart's (A_1_'s) gender	0.19 (0.23)	0.47* (0.20)	−.08 (0.22)	0.40† (0.22)
Dyadic counterpart's (A_1_'s) age group	0.13 (0.18)	0.31† (0.16)	0.04 (0.25)	0.13 (0.24)
Relationship duration	−0.04 (0.03)	0.02 (0.02)	−0.02 (0.03)	0.01 (0.03)
Interaction frequency of dyad	0.17** (0.05)	−0.04 (0.05)	0.20 (0.05)	−0.02 (0.05)
Job level (B_1_)	0.02 (0.16)	−0.05 (0.14)	−0.14 (0.16)	−0.16 (0.16)
Firm tenure (B_1_)	0.01 (0.02)	−0.01 (0.02)	0.00 (0.02)	−0.01 (0.02)
Industry experience (B_1_)	0.00 (0.01)	0.01 (0.01)	−0.01 (0.01)	0.01 (0.01)
Division (B_1_)	−0.27 (0.23)	0.03 (0.20)	−0.35 (0.23)	−0.01 (0.23)
Project (B_1_) (1 = primary, 2 = secondary)	−0.05 (0.14)	−0.13 (0.13)	−0.05 (0.14)	−0.14 (0.14)
*Team‐level control variables*				
Average team age group	–	–	0.31 (0.32)	0.31 (0.32)
Project management team size	–	–	0.10 (0.07)	0.11 (0.06)
*Team‐level interaction*				
Team age heterogeneity	–	–	**−1.02** ** (0.33)	**−0.68** *
Dyadic × team heterogeneity	–	–	**1.28** * (0.51)	**1.02** *
*R* ^2^	0.26	0.30	0.29	0.32
Overall *F*‐ratio	3.33**	4.04**	3.15**	3.70**
*N*	189	191	166	168

† *p* < .1;

* *p* < .05;

** *p* < .01. Also controls for border age groups and mixed gender versus all‐male client teams.

**Figure 2:**
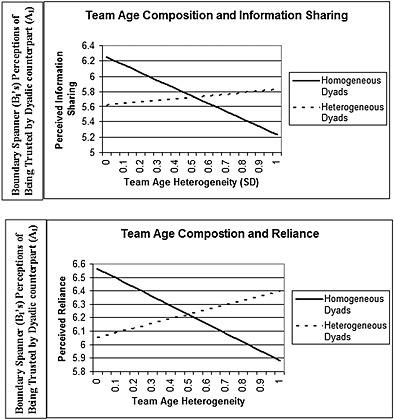
Perception of being trusted and the interaction between dyadic and team age composition

The results for Hypotheses 2 and 3, which reflect an interaction between dyadic and team‐level heterogeneity, are summarized in Table 2 (columns 3 and 4). Hypothesis 2 predicted that boundary spanners (B_1_) in dyadic relationships with clients who are dissimilar in age will perceive more trust from a dyadic client counterpart (A_1_) when the dyad is embedded in a team of clients who are heterogeneous (quadrant III) versus homogeneous in age. We found support for H2 as evidenced by the positive interaction terms on both measures of the perception of being trusted (perceived information sharing, *b* = 1.28, *p* < .05, 95% CI 0.26 ≤ *b* ≤ 2.30; perceived reliance, *b* = 1.15, *p* < .05, CI 0.21 ≤ *b* ≤ 2.09). Because the interaction term is larger in magnitude than the negative effect of team‐level heterogeneity, the net effect of team heterogeneity on the perception of being trusted for dissimilar dyads is positive (slope, *β*_2_ + *β*_3_ = .26 for the perceived information sharing dimension of the perception of being trusted; slope, *β*_2_ + *β*_3_ = .5 for the perceived reliance dimension of the perception of being trusted). Figure 2 illustrates this effect with a dotted black line.

Hypothesis 3 predicted that boundary spanners (B_1_) in dyads whose members are homogeneous in age will perceive less trust from their dyadic client counterpart (A_1_) when the dyad is embedded in a team of clients that are heterogeneous (quadrant II) versus homogeneous in age (quadrant I). Because our interaction equation [Equation (1)] includes a dummy variable and a continuous variable, the *t*‐test for the parameter estimate of the continuous variable represents an accurate test of the significance of the simple slope of the continuous variable for the contrast group (coded as 0, i.e., dyadic age homogeneity) (Aiken et al., 1991). Thus, the negative effect of team heterogeneity on the perception of being trusted within a homogeneous dyad was supported (perceived information sharing dimension, *b* = −1.02, *p* < .01, 95% CI −1.7 ≤ *b* ≤ −0.36; perceived reliance dimension, *b* = −0.65, *p* < .05, 95% CI −1.28 ≤ *b* ≤ −0.02). Figure 2 illustrates this with solid black lines.

The three age heterogeneity variables (dyadic level, team level, and their interaction) explain a significant amount of variance. They account for 15 percent of the explained variance (*R* ^2^) in the perception of being trusted (behavioral reliance) and 21 percent of the explained variance (*R* ^2^) in the perception of being trusted (information sharing dimension).

As illustrated in Figure 2, as team age heterogeneity increased, boundary spanners in heterogeneous dyads moved from the perception of being less trusted by their dyadic counterpart than boundary spanners in homogeneous dyads to the perception of being trusted equally or more than boundary spanners in homogeneous dyads.

We mathematically clarify the results presented earlier by substituting concrete numbers into our regression equations. When the team was homogeneous (team age heterogeneity, *T* = 0), for instance, the difference in the perception of being trusted for boundary spanners (B_1_) in heterogeneous dyads (quadrant IV, where B_1_ is dissimilar from both the dyadic counterpart and all other members of the team) versus homogeneous dyads (quadrant I, where B_1_ is similar to the dyadic counterpart and all other members of the team) was significant and negative at *p* < .05 (−.60 for perceived information sharing and −.48 for perceived reliance). The significance level for this difference is indicated by the *p*‐value for this parameter estimate (see Table 2). At the median value of team age heterogeneity (*T* = 0.45), the difference in perceptions of being trusted for heterogeneous versus homogeneous dyads was close to zero (.03 for perceived information sharing and −.02 for perceived reliance). This difference was not statistically significant as determined by centering the team age heterogeneity variable at its median and re‐running the same regression equations. At the maximum value of team age heterogeneity (*T* = 1), the difference in boundary spanners' (B_1_'s) perceptions of being trusted for heterogeneous versus homogeneous dyads was positive (.75, *p* < .1 for perceived information sharing and .76, *p* < .05 for perceived reliance), thus opposite in sign from the difference when team age heterogeneity equaled zero. Additional sensitivity analyses, reported in Table 3, indicated that the significant negative effect of dyadic age heterogeneity becomes insignificant as team heterogeneity increases above .2. Moreover, as team heterogeneity increases above .75 (for perceived reliance) and above .9 (for perceived information sharing), the coefficient for team heterogeneity with respect to heterogeneous dyads turns positive with increasing levels of statistical significance. For heterogeneous dyads, team heterogeneity above .9 is positively and statistically significantly related to perceived reliance (*p* < .05). Our significant results for age heterogeneity suggest that generational differences did influence the perceived relationships in our consultant–client dyads. Moreover, team heterogeneity may not merely level the playing field with respect to the perception of being trusted in heterogeneous dyads but may have some additional benefits at high levels of team heterogeneity.

**Table 3:** Sensitivity analysis of dyadic heterogeneity on boundary spanner (B_1_'s) perceptions of being trusted by dyadic counterpart (A_1_) at varying levels of team heterogeneity.

Team age heterogeneity centered at …		0.00	0.20	0.25	0.30	Median 0.45	0.75	0.85	0.90	0.92	0.95	1.00
DV = perceived reliance	b_1_ (dyadic heterogeneity)	**−0.483** *	**−0.369** *	**−0.304** †	−0.239	−0.021	**0.45** †	**0.45** †	**0.45** †	**0.762** *	**0.662** *	**0.762** *
	(*SE*)	(0.205)	(0.167)	(0.162)	(0.162)	(0.188)	(0.265)	(0.265)	(0.265)	(0.368)	(0.334)	(0.368)
DV = information sharing	b_1_ (dyadic heterogeneity)	**−0.597** **	**−0.328** †	−0.260	−0.193	0.034	0.42	0.55	**0.617** †	**0.644** †	**0.685** †	**0.752** †
	(*SE*)	(0.221)	(0.170)	(0.166)	(0.166)	(0.172)	(0.292)	(0.335)	(0.357)	(0.366)	(0.380)	(0.403)

† *p* < .1;

* *p* < .05;

** *p* < .01.

## Discussion

On non‐routine, non‐hierarchical projects, being trusted and receiving relevant information from demographically dissimilar team members is critical for a boundary spanner's own competent performance (Wageman, 1995). This study sought to expand our understanding of contextual influences on interpersonal trust. We examined team generational age diversity as an important influence on the perception of being trusted within dyads that are embedded within heterogeneous client teams. We found that the generational diversity of a boundary spanner's client team mattered but that its effect on demographically similar and dissimilar dyads diverged. Even after controlling for emotional closeness and interaction frequency, the generational age diversity of a boundary spanner's client team had a significant negative effect on the boundary spanner's perception of being trusted by a demographically similar client. Although past research has found that similar group membership is associated with greater perceived trustworthiness (Brewer & Brown, 1998), and we found that demographic similarity was positively related to the perception of being trusted by one's dyadic counterpart, our results suggest that team heterogeneity undermines this effect. In contrast, for heterogeneous dyads, team generational age heterogeneity increased boundary spanners' perception of being trusted and included. In this case, team heterogeneity mitigated boundary spanner perceptions of being less trusted by a dissimilar dyadic counterpart.

### Contributions to theory

As the age diversity of the workforce has grown and most organizations have found themselves faced with managing a multigenerational workforce, scholarly interest in age diversity also has increased (Avery et al., 2007; Beatty & Visser, 2005; Fullerton & Toossi, 2001; Joshi et al., 2010; Kunze et al., 2011; Lyons & Kuron, 2014). This study focuses on the impact of a team's generational diversity on the perception of being trusted. The perception of being trusted is important for team members engaged in interdependent tasks because it influences how team members respond to one another and how they interpret each other's behavior. The perception of being *less* trusted not only leads individuals to interpret ambiguous behavior as having sinister intent (Kramer, 1994) but also leads individuals to implement costly safeguards and monitoring strategies to deal with unexpected problems and contingencies rather than costless trust‐based strategies. The perception of being *less* trusted also has organizational impact because it is related to performance and organizational citizenship behavior (Lau & Lam, 2007, 2008; Salamon & Robinson, 2008).

This study contributes to the literature on trust in two ways. First, we unpack how a team's demographic composition influences a boundary spanner's perceptions of dyadic trust. In doing so, we have responded to Rousseau et al.'s (1998) call to shift “the focus away from the interpersonal and toward contextual influences on trust.” (p. 435)—a call echoed by Fulmer and Gelfand (2012). Using social categorization theory and “bonding” theories of social capital, we explicated how demographic composition at the team level differentially influences the perception of being trusted in homogeneous and heterogeneous dyads enmeshed within heterogeneous client teams versus homogeneous client teams. We argued that relative to the level of trust typically available in a heterogeneous versus a homogeneous dyad, the social categorization processes associated with team‐level heterogeneity provide boundary spanner–client dyads with a different pattern of trust ties to other client team members and, thereby, a different level of goodwill and different ability to mobilize additional cooperation from these other team members. Thus, our framework explains why team generational heterogeneity forms a positive context rich in goodwill and cooperation for dissimilar boundary spanner–client dyads embedded within the team (quadrant III, Figure 1) but generates an environment poor in goodwill and cooperation for demographically similar dyads (quadrant II, Figure 1). Our findings are consistent with this argument.

Second, we contribute to the understanding of how boundary spanners' perceptions of being trusted across boundaries are influenced by the demographic composition of a client organization. Studies of interorganizational boundary spanners often use relationship duration as a proxy for trust (e.g., Baker, Faulkner, & Fisher, 1998; Kalnins & Mayer, 2004), without considering the demographic context of the client organization. Some interorganizational researchers examine trust directly (e.g., Perrone et al., 2003; Zaheer, McEvily, & Perrone, 1998); however, these studies do not focus on the contextual influence of the demographic composition of client organizations. Our study showed that after controlling for relationship duration and interaction frequency, team‐level demographic heterogeneity could reduce or increase boundary spanners' perceptions of being trusted at the dyadic level. Interestingly, this suggests that for our boundary spanners out‐group, organizational membership did not eliminate the influence of demography on perceptions of being trusted.

This study also has two implications for the diversity literature. First, although inclusion has typically been investigated within organizations (Roberson, 2006; Shore et al., 2011), we suggest that the benefit of cross‐boundary teams depends upon the experience of inclusion. In the language of Shore et al.'s (2011) theory of inclusion, boundary spanners need to retain the uniqueness that helps them contribute to performance gains; yet, they also need to feel accepted and trusted enough to interact in ways that the team can benefit from their unique contributions. Our study suggests that perceptions of inclusion—that others accept, will rely on, and share information with you—may develop at multiple levels of organizational analysis simultaneously. Not only may team composition influence dyadic experiences as we found in our study, but demographic composition at the department or organizational levels may also influence dyadic experiences. Thus, future research would benefit from looking at how demographic composition at different levels of the organization may support or undermine the influences of demography at other levels, thus jointly influencing how perceptions of trust and inclusion unfold.

Second, although the literature on demography examines the influence of team demographic heterogeneity with respect to individual members' experiences of the team context and with respect to team‐level outcomes (e.g., Chatman & Flynn, 2001; Chatman et al., 1998; Flynn et al., 2001), less attention has been paid to the influence of team demography on team members' perceptions of their homogeneous dyadic relationships embedded within their teams (Joshi & Roh, 2009; Joshi et al., 2011). Similarly, although the demography literature on faultlines has contributed a great deal to unpacking the influence of subgroups on team performance (e.g., Lau & Murnighan, 1998, 2005; Polzer, Crisp, Jarvenpaa, & Kim, 2006; Rico, Molleman, Sánchez‐Manzanares, & Van der Vegt, 2007), scholars in this area have paid less attention to the relational experiences within these homogeneous subgroups (cf. Lau & Murnighan, 2005). If, as our study suggests, members of homogeneous dyads and larger homogeneous subgroups embedded in diverse teams perceive that they are less trusted by one another than they would perceive if they were embedded in homogeneous teams, then the relationships among subgroup members may also impact the cohesiveness and performance of these larger teams.

For example, demographically heterogeneous teams have been associated with process losses, such as increased conflict, lower satisfaction, and reduced face‐to‐face interaction (Ancona & Caldwell, 1992; Chatman et al., 1998; Williams & O'Reilly, 1998, for review). Our results suggest that on diverse teams, this reduced capability for teamwork may stem not only from the lower quality of relationships among dissimilar team members but also from the resultant lower quality of relationships among similar team members. Thus, team diversity may have a complex influence that not only affects the relationships among dissimilar others but also negatively affects the relationships among similar team members.

In our model, we have assumed that the quality of dyadic relationships within teams varies (Uhl‐Bien, 2006), that client team members share leadership of the team (Carson, Tesluk, & Marrone, 2007; Denis, Langley, & Sergi, 2012; Pearce, 2004), and that team demography influences each similar or dissimilar boundary spanner–client dyad in a similar way because of the structural similarity of the relationship of each client to the boundary spanner. However, future research would benefit from empirically examining this relationship and also from looking at the effect of the variance in perceptions of being trusted across multiple dyads within a team on outcomes such as prosocial behavior.

### Implications for practice

Generational stereotypes exist. According to a 2011 poll by the Society for Human Resources, younger workers grumbled that older worker were resistant to change and had a tendency to micromanage, whereas older workers criticized younger workers' informality and need for supervision (SHRM, 2011). Not only do tips for managing intergenerational conflict appear regularly in the popular press (e.g., Psychology Today and AARP), firms that specialize in intergenerational conflict are springing up (e.g., http://www.johnsontraininggroup.com/). Companies ranging from Wal‐mart and Pepsi Co to the American Academy of Nurses have hired consultants to help them manage their multigenerational workforces. Some companies such as Ernst & Young, Citibank, and CVS/pharmacy have implemented polices with the goal of attracting and retaining multiple generations of workers, whereas others such as Time Warner, Cisco, and Booz·Allen have implemented intergenerational mentoring (Hewlett et al., 2009).

Our study suggests that the perception of intergenerational differences can influence the degree to which boundary spanners feel trusted in a dyad and that the generational composition of the counterpart team makes a difference. Understanding that moderate levels of team generational diversity seem to level the playing field for both similar and dissimilar boundary spanner–counterpart dyads provides an opportunity for managers to focus on trust‐building with all team members working with boundary spanners. Managers, who recognize that team building is not just about helping demographically dissimilar individuals build strong relationships but about helping everyone develop strong relationships in a more socially complex environment, will be able to approach culture change and skills training in a way that communicates the value for all team members. For example, team cultures that emphasize psychological safety (Edmondson, 1999) and perspective taking (Fehr & Gelfand, 2012; Williams, 2011, in press) allow individuals to build trust in contexts that encourage risk taking and reduce individuals' tendency to associate blame and distrust with setbacks.

In addition, solo boundary spanners such as founders of high‐tech start‐ups or social entrepreneurs leading non‐profits may need to understand the potential challenges of building trust with teams of individuals from established corporations. Because established corporations are forming alliances with start‐ups and non‐profit organizations at an increasing rate (Bhanji & Oxley, 2013; Henisz, Dorobantu, & Nartey, 2014; Plexus Consulting Group, LLC, 2008; Yaziji & Doh, 2009), the ability to establish trust across organizational and generational boundaries is likely to become increasingly important. For example, as part of a corporate responsibility initiative, Partners in Learning, Microsoft Corporation has over 100 staff members working with local firms and non‐profit organizations such as the education‐related NGO SchoolNet South Africa, the private IT firm Menhaj Educational Technologies, and non‐profit universities, to improve curriculum and teacher development around the globe (Bhanji & Oxley, 2013). These different partners may vary greatly in the generational diversity of the team from the partner organization. Corporate boundary spanners as well as firm founders and non‐profit directors may first need to understand the potential sources of generational misunderstandings and conflict that may undermine their dyadic relationships and then use relational strategies such as perspective taking and threat regulation to actively build interpersonal trust (Williams, 2007, 2011, in press).

Finally, our results suggest that similar boundary‐spanner dyads in homogeneous teams perceive that they are the most trusted. Still, managers and team leaders may want to make sure that boundary spanners do not take advantage of this trust (Skinner, Dietz, & Weibel, 2014) and that creative and conflicting ideas are explored, not silenced.

### Limitations

The study's findings should be considered in light of its limitations. First, our study used self‐report survey methodology, which is appropriate for answering questions about individuals' internal states such as perceptions of being trusted. Common method bias is unlikely to have affected the relationship between demographic heterogeneity, which was calculated from perceptions of visible demographic variables, and the perception of being trusted, an introspective variable (Evans, 1985; Harrison, McLaughlin, & Coalter, 1996; Spector, 2006). In addition, Monte Carlo studies strongly suggest that the significance of interaction effects, which are central to Hypotheses 2 and 3, cannot be attributed to common method variance (Evans, 1985; Harrison et al., 1996).

Second, although we examined two divisions/profit centers that focused on different types of client projects, all respondents in the sample were from the same firm. This sample allowed us to hold constant the influence of firm reputation on participants' initial perception of being trusted, but it may limit our ability to generalize. Our findings, however, are likely to be applicable to many professional service projects, at the very least, and may very well apply to a variety of knowledge creation and knowledge transfer alliances.

Third, because our study analyzes archival data on mid‐level to high‐level professionals, the generational diversity that we investigated included Traditionalists, Baby Boomers, and Gen‐Xers, but not Millennials. Given that Millennials would only increase the generational diversity in a data set, and intergenerational conflict is likely to increase with the number of generations interacting, our study may reflect a conservative test of our hypotheses. Further, the likelihood that our sample represents a conservative test is consistent with Lyons and Kuron's (2014) review article that indicates that recent studies that include more generations have found an even greater number of significant differences in work values than studies with fewer generations represented (e.g., Bristow, Amyx, Castleberry, & Cochran, 2011; Gursoy, Chi, & Karadag, 2013; and respectively Jurkiewicz & Brown, 1998; Jurkiewicz, 2000).

Fourth, our study investigated direct effects of generational age diversity on perceptions of being trusted rather than mediating effects. We established that trust perceptions of a dyad member are affected by generational diversity at both the dyadic and team levels. We argued that social categorization processes that influence social capital at the team level sometimes reinforce and sometimes disrupt social categorization‐based processes at the dyadic level. The direct effects shown in this study provide a foundation for future work that investigates the formation of trust ties in diverse cross‐boundary dyads and teams. We hope that this study has laid the groundwork for future research that can build on the theoretical underpinnings of our arguments and on our insight that dyadic relationships can be influenced by social categorization‐based processes occurring simultaneously at multiple levels of analysis. Such research could take our work farther by examining mediating processes that are influenced by team members' trust ties to the boundary spanner and that influence how trusted boundary spanners feel in their dyadic relationships with team members. Possible mediating processes include psychological mechanisms (e.g., changes in positive affect and perceived trustworthiness) and behavioral mechanisms (e.g., changes in reliance and helping behavior).

Finally, this study used broad generational age ranges to capture demographic differences in age. Individuals, especially those at our generational cutoff points, might consider themselves more similar to others in an adjacent generation. This phenomenon would add error to our analyses and reduce the likelihood of finding significant results. In our analyses, we include the age (in years) of our consultant boundary spanners as well as dummy variables for border age groups to control for this age‐related phenomenon.

## Conclusion

This article explored a boundary spanner's team of counterparts as a context that is likely to influence the perception of being trusted in dyadic cross‐boundary relationships. We argued that the demographic dissimilarity of boundary spanners from their client team members can have opposite implications for dyadic relationships depending upon the demographic similarity of the dyad. We found that diversity among client team members from another organization undermined the perception of being trusted within demographically homogeneous dyads while it increased the perception of being trusted within heterogeneous dyads. Using social categorization theory, we provided insight into how boundary spanners' perception of interpersonal trust within a dyad may be enhanced or constrained by the demographic heterogeneity of their cross‐boundary teams.

## Appendix

**Table A1:** Dichotomous client team‐level heterogeneity and boundary spanner (B_1_'s) perceptions of being trusted by dyadic counterpart (A_1_).^a^

	Perceived information sharing	Perceived reliance
	H2, H3	H2, H3
Dyadic age heterogeneity (1 = heterogeneous, 0 = homogeneous)	−0.73** (0.22)	−0.57** (0.22)
Emotional closeness	0.47** (0.10)	0.36** (0.09)
Interaction frequency of dyad	0.19** (0.06)	−0.02 (0.05)
*Team‐level interaction*		
Team age heterogeneity (1 = heterogeneous, 0 = homogeneous)	−0.66** (0.20)	−0.42* (0.19)
Dyadic × team heterogeneity	**0** **.84** ** (0.32)	**0.68** * (0.29)
∆*R* ^2^ (with interaction term)	0.034**	0.026*
*R* ^2^	0.29	0.31
Overall *F*‐ratio	2.90**	3.20**
*N*	168	170

*Note:* Bold numbers test hypotheses.

a All control variables reported in Table 2 were also used in the analyses reported in Table 2.

† *p* < .1;

* *p* < .05;

** *p* < .01.
